# Fecal Galectin-3: A New Promising Biomarker for Severity and Progression of Colorectal Carcinoma

**DOI:** 10.1155/2018/8031328

**Published:** 2018-04-04

**Authors:** Milan Jovanovic, Nevena Gajovic, Natasa Zdravkovic, Marina Jovanovic, Milena Jurisevic, Danilo Vojvodic, Veljko Maric, Aleksandar Arsenijevic, Ivan Jovanovic

**Affiliations:** ^1^Department of Abdominal Surgery, Military Medical Academy, Belgrade, Serbia; ^2^Center for Molecular Medicine and Stem Cell Research, Faculty of Medical Sciences, University of Kragujevac, Kragujevac, Serbia; ^3^Department of Internal Medicine, Faculty of Medical Sciences, University of Kragujevac, Kragujevac, Serbia; ^4^Department of Pharmacy, Faculty of Medical Sciences, University of Kragujevac, Kragujevac, Serbia; ^5^Institute for Medical Research, Military Medical Academy, Belgrade, Serbia; ^6^Department of Surgery, Faculty of Medicine Foca, University of East Sarajevo, Sarajevo, Bosnia and Herzegovina

## Abstract

**Background and Objectives:**

The aim of the study was to determine systemic and fecal values of galectin-3 and pro- and anti-inflammatory cytokines in patients with CRC and the relationship with clinicopathological aspects.

**Methods:**

Concentrations of galectin-3, TNF-*α*, TGF-*β*, IL-10, and IL-1*β* were analyzed in samples of blood and stool of 60 patients with CRC.

**Results:**

Systemic concentration of TNF-*α* was significantly lower in patients with severe diseases (advanced TNM stage, nuclear grade, and poor histological differentiation) as in patients with more progressive CRC (lymph and blood vessel invasion, presence of metastasis). Fecal values of anti-inflammatory cytokines TGF-*β* and IL-10 were increased in patients with severe stadium of CRC. Fecal concentration of Gal-3 was enhanced in CRC patients with higher nuclear grade, poor tumor tissue differentiation, advanced TNM stage, and metastatic disease. Gal-3/TNF-*α* ratio in sera and feces had a higher trend in patients with severe and advanced diseases. Positive correlation between fecal Gal-3 and disease severity, tumor progression, and biomarkers AFP and CEA, respectively, was also observed.

**Conclusions:**

Predomination of Gal-3 in patients with advanced diseases may implicate on its role in limiting ongoing proinflammatory processes. The fecal values of Gal-3 can be used as a valuable marker for CRC severity and progression.

## 1. Introduction

Of cancers that affect both men and women, colorectal cancer (CRC) is the fourth leading cause of cancer death in the world [1, 2]. It is the third most common cancer in males and the second in females [1, 2]. Although the distribution of CRC varies widely, more than two-thirds of all cases and more than half of all deaths happen in countries with high human development index (HDI) [3]. Besides the important role of genetic factors such as mutations of oncogenes and tumor suppressor genes and history of CRC in first-degree relatives, environmental factors, such as inflammatory bowel disease, increased body mass index (BMI), red meat intake, cigarette smoking, low physical activity, and low vegetable and fruit consumption, are associated with an increased risk of CRC [4, 5]. CRC metastasizes to the liver and lungs, while bone metastasis often indicates the terminal phase of colon cancer [6]. Despite the fact that around 80% of patients with CRC have primary surgery, about half of the patients already have metastatic lesions primarily in the liver [7, 8]. Surgery, as well as radiofrequency ablation, cryosurgery, chemotherapy, radiation therapy, or targeted therapy, is the most common treatment option for CRC [4]. Although a 5-year survival or stage I–III CRC is pretty good, cancer-related deaths are registered in one-third of patients younger than 65 years old at disease onset [9]. Although the gold standard for CRC diagnosis is a colonoscopy procedure, there is a tendency to use more noninvasive tests such as measurement of different molecules in sera and feces of patients [10]. There has been a sustained interest in the identification of state biomarkers for CRC [11–13]. New markers should contribute to the prediction of prognosis. Recent studies revealed the significance of estimation of fecal markers in the determination and prediction of disease severity [14–16].

Galectin-3 (Gal-3) is a multifunctional *β*-galactoside-binding lectin highly expressed in a variety of inflammatory and epithelial cells [17]. Multiple functions of Gal-3 depend on its location inside the cell or on the cell surface [18]. It is well known that Gal-3 is involved in several biological processes such as cell attachment, cell differentiation and proliferation, embryogenesis, inflammation, cancer invasion, and metastasis [19, 20]. Previous studies revealed the importance of Gal-3 as a prognostic marker in CRC. It is shown that patients with detectable expression of Gal-3 in tumor have more lymph node and distant metastases, frequent venous invasion, and deeper wall invasion in comparison to those with Gal-3-negative cases [21]. Moreover, a recent study revealed that serum galectin-3 and carcinoembryonic antigen (CEA) promote CRC migration and metastasis [22].

The aim of this study was to evaluate systemic and fecal values of Gal-3 and pro- and anti-inflammatory cytokines, as well as their ratios, in patients with CRC and UC and the relationship with clinicopathological aspects of disease. We demonstrate enhanced fecal concentration of Gal-3 in CRC patients with higher nuclear grade, poor tumor tissue differentiation, advanced TNM stage, and metastatic disease, while predomination of Gal-3 over proinflammatory cytokines in patients with advanced TNM stage and metastatic disease. Fecal Gal-3 positively correlates with disease severity (advanced TNM stage, higher nuclear grade, and poor tumor tissue differentiation) and progression (presence of lung/liver metastasis or peritoneal carcinomatosis) and systemic biomarkers AFP and CEA. There was no significant correlation between fecal Gal-3 and clinical and endoscopic scores and histopathological characteristics of affected tissue in patients with ulcerative colitis. These findings indicate Gal-3 as a potential marker of CRC severity and progression.

## 2. Materials and Methods

### 2.1. Ethics Approval

The study was conducted at Center for Gastroenterology, Clinical Center of Kragujevac, and Center for Molecular Medicine and Stem Cell Research, Faculty of Medical Sciences, University of Kragujevac, Serbia. Informed consent was obtained from all subjects. The study was approved by the ethics committee of the Clinical Center of Kragujevac, Kragujevac, Serbia, and Faculty of Medical Sciences, University of Kragujevac, Serbia. All research procedures were made according to the principle of Good Clinical Practice and the Declaration of Helsinki.

### 2.2. Patients

The study included 60 patients with CRC. The diagnosis of CRC was based on endoscopic and histopathological criteria. Patients with no well-defined pathology, no adequate clinical document available, or previously treated with radiation or chemotherapy were excluded from the study. Clinicopathological information for all patients included sex, age, TNM stage, vascular/lymph node invasion, nuclear grade, and differentiation. Blood and stool specimens were collected before the surgery and stored at −80°C. Pathological features were analyzed according to the 2010 American Joint Committee on Cancer (AJCC) classification.

Fifty patients, with a median age of 55 (range, 23–73 years), diagnosed as UC cases, were also enrolled in this study. Diagnosis was made on the basis of established clinical, endoscopic, and histological criteria [23]. The study did not include patients without well-defined pathology, no adequate clinical document available, or with previously diagnosed coexisting cardiopulmonary, renal, hepatic, allergy, and rheumatic disease who were treated with anti-inflammatory drugs. Stool samples were collected before the surgery and stored at −80°C. Clinical activity of disease and endoscopic findings were represented as Mayo clinical/endoscopic subscore, defined as previously described [24–26]. Histological activity was scored according to Geboes Score (GS), considering the presence of architectural changes, neutrophils, eosinophils, crypt destruction, and erosion of the mucous membranes [27].

### 2.3. Measurement of *Galectin-3*, *TNF-α*, *TGF-β*, *IL-10*, and *IL-1β* in Sera and Feces

All samples were collected prior to any therapeutic application. Blood specimens were collected from each studied subject; blood clot was cut and centrifuged for separating the serum; and all serum samples were kept at −80°C before use. Stools (1–10 g) were collected in sterile containers and weighed. They were divided into 1 g aliquot and then emulsified in 5 mL of protease inhibitor cocktail (SIGMA, P83401), diluted 1 : 100, and centrifuged for 5 minutes at 400*g*, at 4°C, as previously described [28, 29]. The supernatant fluid was collected and stored at −80°C until ELISA. Serum and fecal concentrations of cytokines were measured, as described [30], using sensitive enzyme-linked immunosorbent assay (ELISA) kits (R&D Systems, Minneapolis, MN) specific for human cytokines according to the manufacturer's instructions. Briefly, the PVC microtiter plates were coated with capture antibody, overnight. After blocking the remaining protein-binding sites by adding blocking buffer (1% bovine serum albumin in PBS) for 1 hour, serum/fecal samples or standard recombinant Gal-3/TNF-*α*/IL-1*β*/TGF-*β*/IL-10 were added to the plates for 2 hours, followed by application of biotinylated detection antibody for 1 hour at room temperature. After introduction of streptavidin peroxidase for 1 hour, the plates were developed with substrate reagent for 20 minutes. The reaction was stopped by adding 4 mol/L sulfuric acid, and the absorbance was read at 495 nm by a microplate reader. Concentration of the samples was measured by intrapolation from the standard curve made by a series of well-known concentrations as per manufacturer's instruction. Values of measured cytokines are presented as pg/ml of sera and pg/g of feces, respectively. The lower detection limit (sensitivity) of the ELISA kits, for measured cytokines, was galectin-3: 85 pg/ml; TNF-*α*: 5.5 pg/ml; TGF-*β*: 15.4 pg/ml; IL-10: 3.9 pg/ml, and IL-1*β*: 1 pg/ml.

### 2.4. Evaluation of Tumor Markers in Sera

Serum levels of tumor markers alpha-fetoprotein (AFP), carcinoembryonic antigen (CEA), and cancer antigen 19-9 (CA19-9) were determined by chemiluminescence enzyme immunoassay (CLIA) in the central biochemical laboratory of the Clinical Center Kragujevac.

### 2.5. Statistical Analysis

The statistical analyses were performed using SPSS 20.0 software. The results were reported as mean, standard deviation (SD), and standard error (SE). Determination of statistically significant difference between the means of two groups was determined using Student's *t*-test for independent samples if the data had normal distribution or Mann–Whitney *U* test for data without normal distribution. Kruskal–Wallis test was used to determinate statistically significant difference between the means of three groups. Pearson's correlation evaluated the possible relationship between the cytokines and disease severity and progression in patients with CRC. The strength of correlation was defined as negative or positive weak (−0.3 to −0.1 or 0.1 to 0.3), moderate (−0.5 to −0.3 or 0.3 to 0.5), or strong (−1.0 to −0.5 or 1.0 to 0.5). A *p* value of 0.05 was considered statistically significant.

## 3. Results

Sixty adult patients with CRC and fifty with UC were included in this study. There was no significant difference in gender distribution. The average age of all patients with CRC is 64 ± 1 and of patients with UC is 55 ± 1. Clinical and pathologic characteristics of these patients are presented in Table 1. We have assessed concentration of pro- and anti-inflammatory cytokines, as well as Gal-3, and tumor markers (AFP, CEA, and CA19-9) in serum and feces liquid fraction.

### 3.1. Fecal Concentration of Gal-3 Associated to Histopathologic Characteristics of CRC

**Figure 1:**
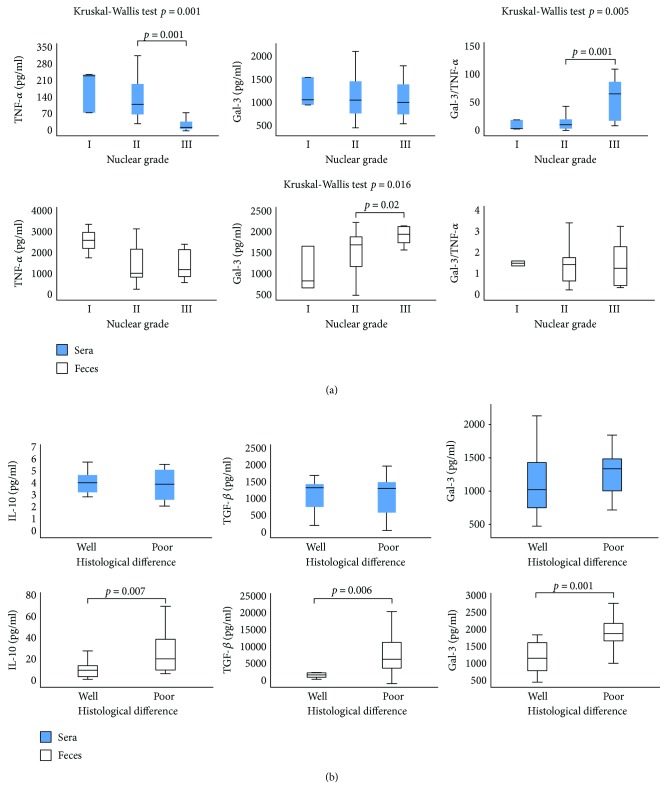
(a) Increased concentration of Gal-3 in feces and Gal-3/TNF-*α* ratio in sera, in patients with a higher nuclear grade of CRC. Patients with CRC were divided into three groups, based on nuclear grades (I, II, and III). Serum and fecal levels of all mentioned biomarkers were determined by ELISA. Gal-3/TNF-*α* ratios were evaluated for each patient, separately. Kruskal–Wallis test was used to determinate statistically significant difference between the means of three groups. (b) Increased concentrations of IL-10, TGF-*β*, and Gal-3 in the feces of patients with poor histological differentiation of CRC. Patients with CRC were divided into two groups, according to histological differentiation rate (well/moderate and poor). Statistical significance was tested by Mann–Whitney Rank Sum test.

Firstly, patients with CRC were classified in four groups based on the nuclear grades of tumor tissue: I, II, III, and IV. This classification was based on the evaluation of the size and shape of the nucleus in tumor cells and the percentage of tumor cells that are in the process of dividing or growing [31]. Recent studies have shown associations between nuclear grading and aggressiveness underscoring the importance of nuclear grading beyond prognostic stratification [31, 32]. We did not estimate nuclear grade IV in any of the CRC patients. Evaluation of systemic levels of the previously defined markers of interest revealed significantly lower level of TNF-*α* in the group of patients with nuclear grade III in comparison to patients with nuclear grade I or II (*p* = 0.001; Figure 1(a)). There were no statistical differences in the serum level of Gal-3 between the defined groups. However, Gal-3/TNF-*α* ratio was significantly higher in the patients with nuclear grade III (*p* = 0.001; Figure 1(a)). There was no significant difference in the fecal level of TNF-*α* between the patients with different nuclear grades of CRC (Figure 1(a)). We noticed significant increment of the fecal level of Gal-3 in the group of patients with nuclear grade III compared to grades I and II (*p* = 0.02), while there was no difference in the Gal-3/TNF-*α* ratio (Figure 1(a)).

Next, patients were divided into two groups according to histological differentiation rate: well/moderate and poor. Well-differentiated and moderately differentiated tumors (well/moderate) were defined as low-grade lesions, whereas poorly differentiated tumors (poor) were defined as high-grade lesions according to the WHO guidelines [33]. Grading was based on the evaluation of the worst area, excluding areas of focal dedifferentiation present at the invasive margin of the tumor [34]. Poorly differentiated tumors have repeatedly been shown to behave more aggressively than well/moderate-differentiated carcinomas in multivariate analysis [34]. We did not find significant differences in the serum level of IL-10, TGF-*β*, and Gal-3 between the defined groups (Figure 1(b)). However, fecal levels of Gal-3 (*p* = 0.001) and anti-inflammatory cytokines IL-10 (*p* = 0.007) and TGF-*β* (*p* = 0.006) were significantly higher in patients with poorly differentiated CRC (Figure 1(b)).

### 3.2. Fecal Gal-3 and Gal-3/TNF-*α* Ratio Associated with TNM System and Lymph and Blood Vessel Invasion

**Figure 2:**
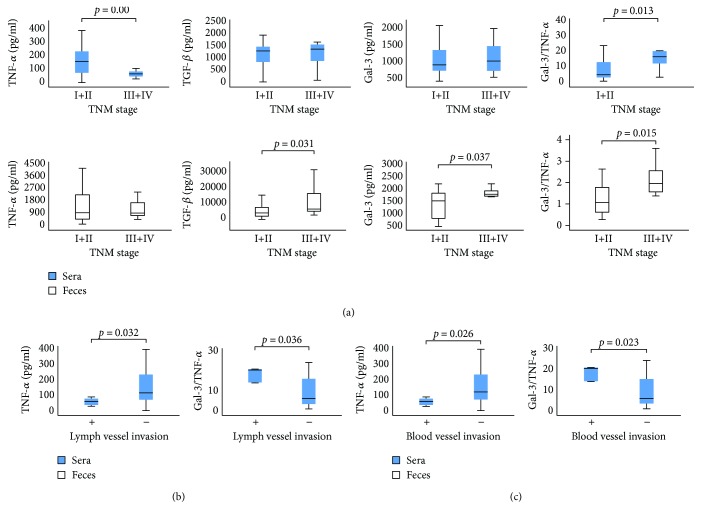
(a) Increased concentration of TGF-*β*, Gal-3, and Gal-3/TNF-*α* ratios in feces in patients with higher TNM stage of CRC. The patients with CRC were divided into two groups, based on the TNM stage (I + II and III + IV). Serum and fecal levels of all mentioned biomarkers were determined by ELISA. Gal-3/TNF-*α* ratio was evaluated for each patient, separately. (b-c) Decreased TNF-*α* and increased Gal-3/TNF-*α* ratio in the sera of patients with detectable lymphatic and blood vessel invasion of CRC. The patients with CRC were divided into two groups, based on the presence of lymphatic/blood vessel invasion (+ and −). Serum levels of all mentioned biomarkers were determined by ELISA. Gal-3/TNF-*α* ratio was evaluated for each patient, separately. Statistical significance was tested by Mann–Whitney Rank Sum test or independent samples *t*-test, where appropriate.

Patients with CRC were divided into two categories on the basis of TNM stage of disease: I + II and III + IV. Patients with TNM stages III + IV revealed significantly lower TNF-*α* in sera in comparison to patients with TNM stages I + II (*p* = 0.006; Figure 2(a)). There were no differences in the serum level of TGF-*β* and Gal-3 (Figure 1(b)). The Gal-3/TNF-*α* ratio was higher in the sera of patients with TNM stages III + IV, but this difference did not reach statistical significance (Figure 2(a)).

As shown in Figure 2(a), CRC patients with higher TNM stages appear to have a higher fecal level of TGF-*β* (*p* = 0.031). There was no difference in the fecal level of TNF-*α* between the defined groups (Figure 2(a)). We noticed a higher fecal level of Gal-3 (*p* = 0.037) as well as Gal-3/TNF-*α* ratio (*p* = 0.015) in patients with TNM stages III + IV (Figure 2(a)).

Further, we divided the patients based on the invasion of lymph and blood vessels, respectively (+ and −), and analyzed their serum levels of biomarkers. TNF-*α* was significantly decreased in patients with detected lymph or blood vessel invasion (*p* = 0.032; *p* = 0.026; Figures 2(b) and 2(c)). Moreover, an increased Gal-3/TNF-*α* ratio in sera was evaluated in patients with detectable lymphatic (*p* = 0.036) and blood vessel invasion (*p* = 0.023; Figures 2(b) and 2(c)).

### 3.3. Liver, Lung, and Peritoneal Metastasis Associated with Higher Fecal Gal-3

**Figure 3:**
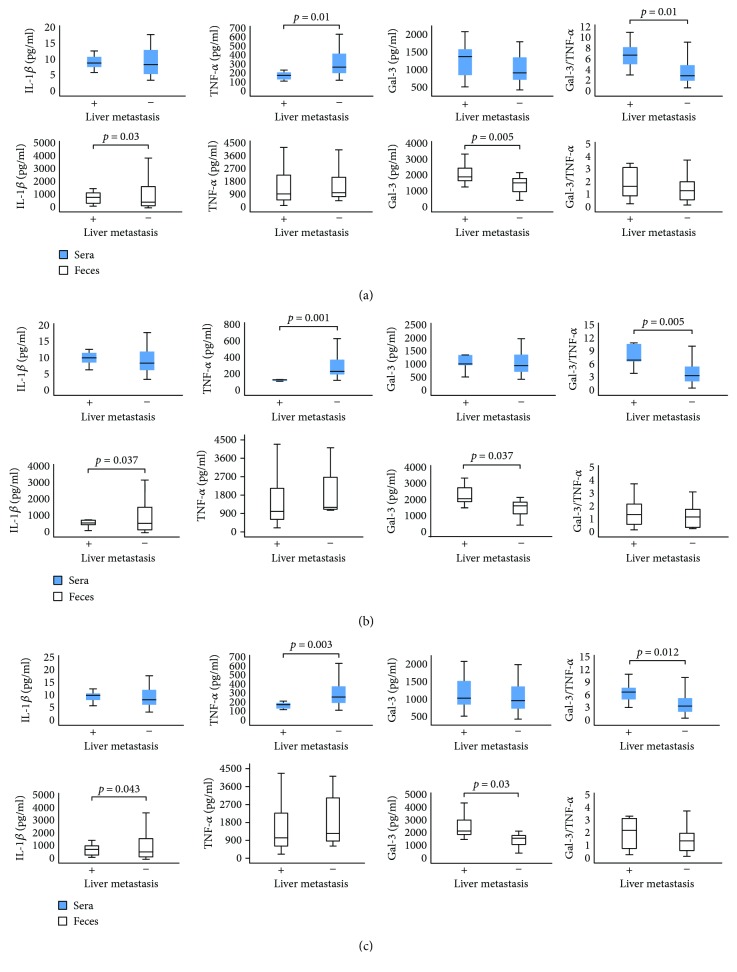
(a) Decreased fecal IL-1*β* and systemic TNF-*α*, while increased fecal Gal-3 and systemic Gal-3/TNF-*α* ratios in patients with detectable liver metastasis. Patients with CRC were divided into two groups, based on the presence of liver metastasis (+ and −). (b) Decreased fecal IL-1*β* and systemic TNF-*α*, while increased fecal Gal-3 and systemic Gal-3/TNF-*α* ratios in patients with detectable lung metastasis. The patients with CRC were divided into two groups, based on the presence of lung metastasis (+ and −). (c) Decreased fecal IL-1*β* and systemic TNF-*α*, while increased fecal Gal-3 and systemic Gal-3/TNF-*α* ratios in patients with detectable peritoneal carcinomatosis. The patients with CRC were divided into two groups, according to the presence of carcinomatosis in peritoneum cavity (+ and −). The serum levels of all mentioned biomarkers were determined by ELISA. Gal-3/TNF-*α* ratio was evaluated for each patient, separately. The statistical significance was tested by Mann–Whitney Rank Sum test.

Next groups of patients with CRC were made according to the presence of lung/liver metastasis or peritoneal carcinomatosis, respectively, and analyzed for values of Gal-3 and other mediators of interest. There were no differences in the systemic concentrations of IL-1*β* and Gal-3 between the patients with and without metastasis/carcinomatosis (Figure 3). Lower TNF-*α* but a higher Gal-3/TNF-*α* ratio was found in the sera of patients with detectable liver metastasis, lung metastasis, or peritoneal carcinomatosis, in comparison to patients without metastasis/carcinomatosis (*p* < 0.05 all, Figure 3). Feces concentration of IL-1*β* was significantly lower while the level of Gal-3 was significantly higher in patients with metastasis/carcinomatosis. There were no differences in the feces levels of TNF-*α* and neither in the Gal-3/TNF-*α* ratio between the defined groups.

### 3.4. Fecal Gal-3 Concentration Significantly Correlated with CRC Severity, but Not with UC Severity

The relationship between fecal Gal-3 and the clinicopathological parameters of patients with CRC and UC, respectively, were summarized in Table 2. An analysis revealed a positive correlation between fecal galectin-3 and parameters and markers of disease severity and progression. There is a moderate positive correlation between fecal galectin-3 and nuclear grade (*r* = 0.358; *p* = 0.025), histological type (*r* = 0.543; *p* = 0.001), TNM stage (*r* = 0.339; *p* = 0.035), presence of liver metastasis (*r* = 0.406; *p* = 0.004), presence of lung metastasis (*r* = 0.303; *p* = 0.036), presence of peritoneal carcinomatosis (*r* = 0.420; *p* = 0.003), and tumor markers AFP (*r* = 0.438; *p* = 0.002) and CEA (*r* = 0.308; *p* = 0.049). We did not find a correlation between systemic galectin-3 and the same parameters (data not shown). Further, we found no correlation between fecal Gal-3 and parameters and markers of UC severity (endoscopic score, clinical score, crypt destruction, erosion of the mucous membranes, architectural changes, neutrophil infiltration, and eosinophil infiltration; Table 2).

**Figure 4:**
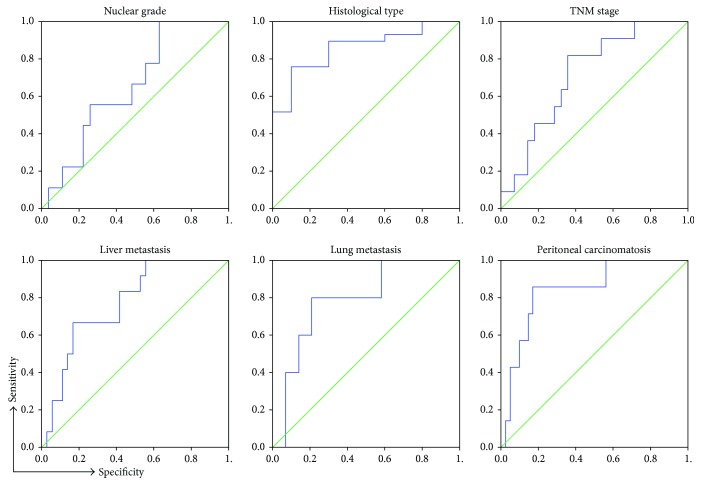
Specificity and sensitivity of fecal Gal-3. ROC curves illustrate the specificity and sensitivity of fecal Gal-3 comparing nuclear grade, histological type of tumor tissue (well/moderate versus poor differentiated), TNM stage (I + II versus III + IV), presence of liver metastasis (+ versus −), lung metastasis (+ versus −), and peritoneal carcinomatosis (+ versus −).

Analysis of Receiver Operating Characteristic (ROC) curves of fecal galectin-3 for various stages and parameters of CRC found that galectin-3 level in feces could predict disease severity (Figure 4). The analysis showed that Gal-3 can be a valuable marker for distinguishing the nuclear grade (sensitivity 88.7%, specificity 83.8%) and histological type of CRC (sensitivity 88.7%, specificity 83.8%), TNM stage (sensitivity 88.7%, specificity 83.8%), presence of liver metastasis (sensitivity 88.7%, specificity 83.8%), lung metastasis (sensitivity 88.7%, specificity 83.8%), and peritoneal carcinomatosis (sensitivity 88.7%, specificity 83.8%). The optimal cutoff value estimated for Gal-3 that allows the discrimination of stages of CRC progression was 1958.82 pg/g. For this cutoff, we determined sensitivity to be 81.8% and specificity 60.7%.

## 4. Discussion

Disease severity depends of cytokine milieu that dominates in a tumor environment. Previous studies established that presence of CD4+ Th1 cells and cytotoxic CD8+ T cells (CTLs) in tumor microenvironment presents positive prognostic sign, while presence of Treg and Th2/Th17 cells indicates lower survival of patients with CRC [35, 36]. Predomination of proinflammatory TNF-*α*/IFN-*γ*-producing Th1 cells, besides enhancing CTL activity, also facilitates innate antitumor mechanisms and associates with the absence of metastatic invasion, tumor recurrence, and increased survival of patients with CRC [37–39]. On contrary, Th2-polarized CD4+ T cells produce IL-4, IL-10, and IL-13, enhancing local humoral immunity and suppressing Th1 immune response [36]. In line with this phenomenon, previous studies have shown that patients with bladder and colorectal cancer have decreased proportions of IFN-*γ*/IL-2-producing Th1 cells, while increased proportions of IL-4/IL-10-producing Th2 cells, in peripheral blood [40, 41]. Presence of immunosuppressive TGF-*β*/IL-10-producing innate/adaptive immune cells correlates with advanced disease and poor prognosis [35]. In the present study, we analyzed concentration of pro- and anti-inflammatory cytokines in sera and feces of patients with CRC. Systemic values of proinflammatory cytokine TNF-*α* were significantly lower in patients with severe disease (TNM stages III and IV, poor histological differentiation, and nuclear grade III) (Figures 1 and 2). Moreover, systemic TNF-*α* was significantly lower in patients with more progressive CRC (lymph and blood vessel invasion and presence of metastasis in the liver, lung, and peritoneal carcinomatosis; Figures 2 and 3). Local values of cytokines in liquid fraction of feces have shown predomination of anti-inflammatory cytokines TGF-*β* and IL-10 in patients with severe stadium of CRC (TNM stages III and IV, poor histological differentiation) as well as lower level of proinflammatory IL-1*β* in patients with a more progressive disease (presence of metastasis in the liver, lung, and peritoneal carcinomatosis; Figures 1, 2, and 3). These results are in line with our and other previous studies claiming that serum levels of IL-10 and TGF-*β* were increased in patients with CRC in comparison to healthy controls and that CRC patients with worse prognosis had increased systemic concentration of IL-10 compared to patients with better prognosis [11, 42].

Gal-3 is involved in various biological and pathological processes, such as cell proliferation and differentiation, cell and extracellular matrix interactions, metastasis, and regulation of apoptosis [43, 44]. Earlier studies have investigated possible linkage between Gal-3 and CRC. Immunohistochemical staining confirmed that colon cancer with detected Gal-3 was significantly larger, with deeper invasion to the colonic wall and with poor histological differentiation [25, 45]. Others showed that Gal-3 upregulation correlated with tumor progression and predicted shorter survival of CRC patients [46]. In contrast, some studies showed decreased Gal-3 levels in CRC progression and that there was no significant correlation between galectin-3 and tumor staging of colon cancer [13, 47]. These findings highlight the importance of extracellular Gal-3 in CRC biology. Recently, studies point on significance of measuring biomarkers in feces [14, 15]. In this way, proteins and molecules produced by intestinal mucosa are measured, which reflect condition in bowels [48]. Recent studies have shown elevated fecal values of M2 pyruvate kinase, fecal calprotectin, and iFOBT in CRC and suggested for screening high-risk groups for CRC [49]. Today, researchers are testing diagnostic accuracy of different fecal markers in the detection of cancerous lesions in the colon, in order to find the most accurate for CRC screening. To our knowledge, this is the first study testing fecal Gal-3 for detection of severe and progressive forms of CRC. We have not found differences in the systemic level of Gal-3 between different stages of colorectal cancer. However, significantly increased fecal level of Gal-3 was detected in patients with a more severe stage of CRC (poor histological differentiation and higher nuclear grade and TNM stages III and IV; Figures 1 and 2). Moreover, fecal Gal-3 was significantly increased in patients with lymph and blood vessel invasion and with presence of metastasis in the liver, lung, or peritoneal carcinomatosis, respectively (Figures 2 and 3).

As it is well known that the ratio of counterregulatory cytokines is a reliable marker of the disease process, we have analyzed the ratio of Gal-3 with pro- and anti-inflammatory cytokines. There were no differences in the ratio of Gal-3 and TGF-*β*, IL-10, and IL-1*β*. However, we noticed predomination of Gal-3 over proinflammatory cytokine TNF-*α* in sera and feces of patients with more severe and progressive stadium of CRC (Figures 1, 2, and 3). Based on these findings, we believe that the Gal-3/TNF-*α* ratio could be a predictor for the advanced stages of colorectal cancer. There are few possible mechanisms that can explain the potential role of Gal-3 in CRC progression. Firstly, Gal-3 can exert anti-inflammatory effect. The role of Gal-3 in the onset, progression and resolution of inflammation is well established [19]. It is well known that during inflammation, reactive oxygen species and other toxic products accumulate in cell and induce production of advanced glycation end products (AGEs) [50]. Further, AGEs bind to receptor (RAGE) thus facilitating local inflammation [51]. Gal-3 inhibits AGE-RAGE pathway and subsequently suppresses RAGE-induced inflammation in tissue [52]. Another possible mechanism of act of Gal-3 is direct inhibition of cellular immune response, by inhibiting interaction between T cells and antigen-presenting cells [53]. Others claimed that extracellular galectin-3 induces apoptosis of CD4 or CD8 T cells in the tumors and can suppress IFN-*γ* secretion of human cytotoxic T cells in tumor [54]. We are first to describe prevalence of Gal-3 over TNF-*α* in stool of patients with severe and progressive forms of CRC (Figures 2 and 3).

Besides these roles in immunomodulation, Gal-3 can facilitate migration of colon cancer cells through the K-Ras-Raf-Erk1/2 pathway [55]. Gal-3 can exert clustering of integrins, leading to cell motility, while binding to mucin-1, a molecule that dominates in gastrointestinal environment and facilitates transendothelial invasion [56].

Further in this study, we envisage the possible role of fecal galectin-3 as a biomarker in preceding disease severity and progression. We obtained a positive correlation between the fecal Gal-3 and disease severity (advanced TNM stage, higher nuclear grade, and poor tumor tissue differentiation; Table 2 and Figure 4). Moreover, the fecal level of Gal-3 is in a positive correlation with more progressive CRC (presence of lung/liver metastasis or peritoneal carcinomatosis; Figure 4). We have also shown a strong positive correlation between the fecal Gal-3 and biomarkers AFP and CEA, respectively (Table 2). Interestingly, we did not find a correlation of serum Gal-3 with the same parameters and markers of the disease severity. Also, values of Gal-3 in feces are about two to three times higher than those in sera, what makes measurement in feces a more sensitive method. In CRC, AFP and CEA have been used as reliable tumor markers for monitoring tumor progression. Recent studies demonstrated that Gal-3 interacts with CEA, promoting colorectal cancer cell migration, adhesion, and subsequent metastasis [22, 57]. Analysis on UC patients revealed that fecal Gal-3 does not correlate with the endoscopic and clinical Mayo score, as well as with histopathological parameters of the affected tissue, and is not suitable for evaluation of severity of UC (Table 2).

Analysis of Receiver Operating Characteristic (ROC) curves of Gal-3 and disease parameters and markers for CRC revealed that Gal-3 could predict an advanced TNM stage, higher nuclear grade, and poor tumor tissue differentiation as well as the presence of lung/liver metastasis or peritoneal carcinomatosis, at good sensitivity and specificity. According to our findings, fecal Gal-3 could be a valuable marker for CRC severity and progression.

## 5. Conclusions

In summary, increased local values of Gal-3, reflected through a higher fecal concentration, in CRC patients with a higher nuclear grade, poor tumor tissue differentiation, and advanced TNM stage of disease, may be considered as a sign of the tumor's malignant progression and, consequently, of a poor prognosis for patients. Predomination of Gal-3 over proinflammatory cytokine TNF-*α*, in patients with advanced and progressive disease, may implicate on immunomodulatory role of Gal-3 in limiting ongoing proinflammatory processes and preventing potent antitumor immune response. This phenomenon favors tumor immune escape and long-range dissemination of tumoral cells (metastasis). Furthermore, the fecal values of Gal-3 can be used as a valuable marker for CRC severity and progression and not for UC severity. These observations support the idea that Gal-3 may contribute to the immune privilege of tumors by modulating local immune response and point on possible role of fecal Gal-3 as a state and progression marker of CRC and its potential use as a therapeutic target.

## Figures and Tables

**Table 1:** Baseline characteristics of patients.

	Number
Colorectal carcinoma (CRC)	
Gender (male/female)	35/25
Age (mean (range))	64 (50–82) years
Site (P/D/R)	15/34/11
Nuclear grade (I/II/III)	8/37/15
Histological differentiation rate (well/moderate/poor)	11/33/16
Stage (TNM: I/II/III/IV)	42/0/16/12
Necrosis (well/moderate/absent)	16/44/0

Ulcerative colitis (UC)	
Gender (male/female)	29/21
Age (mean (range))	55 (23–73) years
Endoscopic score (0/I/II/III)	0/26/16/8
Clinical score (0/I/II/III)	0/28/14/8
Crypt destruction (0/I/II/III)	4/25/9/12
Erosion of the mucous membranes (0/I/II/III/IV)	12/13/6/9/10
Architectural changes (0/I/II/III)	0/23/14/13
Neutrophil infiltration (0/I/II/III)	5/19/8/18
Eosinophil infiltration (0/I/II/III)	10/16/13/11

P: proximal colon; D: distal colon; R: rectum.

**Table 2:** Correlation between the fecal level of Gal-3 and parameters of disease severity and progression in patients with CRC and UC. Statistical significance was tested by Spearman correlation coefficient.

Variables	Gal-3	
	Spearman's rho	*p* value
CRC		
Nuclear grade	0.358	0.025
Histological type	0.543	0.001
Dukes stage	0.339	0.035
Liver metastasis	0.406	0.004
Lung metastasis	0.303	0.036
Peritoneal carcinomatosis	0.420	0.003
AFP	0.438	0.002
CEA	0.308	0.049
CA19-9	0.254	0.088

UC		
Endoscopic score	0.187	0.172
Clinical score	0.091	0.511
Crypt destruction	0.193	0.159
Erosion of the mucous membranes	0.170	0.215
Architectural changes	0.100	0.468
Neutrophil infiltration	0.107	0.435
Eosinophil infiltration	0.035	0.799
